# Empowering frail older adults: multicomponent elastic-band exercises and BCAA supplementation unleash physical health and preserve haematological biomarkers

**DOI:** 10.3389/fspor.2023.1171220

**Published:** 2023-08-30

**Authors:** Adriana Caldo-Silva, Guilherme E. Furtado, Matheus Uba Chupel, Rubens Vinícius Letieri, Rafael Santos Neves, Fábio Direto, Marcelo P. Barros, André L. L. Bachi, Ander Matheu, Faber Martins, Alain Massart, Ana Maria Teixeira

**Affiliations:** ^1^ Research Centre for Sport and Physical Activity, CIDAF, Faculty of Sport Science and Physical Education, University of Coimbra, Coimbra, Portugal; ^2^ Polytechnic Institute of Coimbra, Applied Research Institute, Rua da Misericórdia, Lagar dos Cortiços-S, Martinho do Bispo, Coimbra, Portugal; ^3^ Research Centre for Natural Resources Environment and Society (CERNAS), Polytechnic Institute of Coimbra, Bencanta, Coimbra, Portugal; ^4^ Biological Sciences Platform- Hurvitz Brain Sciences, Sunnybrook Research Institute, Toronto, ON, Canada; ^5^ Multidisciplinary Research Nucleus in Physical Education (NIMEF), Physical Education Department, Avenida Nossa Senhora de Fátima, Federal University of North of Tocantins (UFNT), Tocantinópolis, Brazil; ^6^ Institute of Physical Activity Sciences and Sports (ICAFE), Interdisciplinary Program in Health Sciences, Cruzeiro do Sul University, São Paulo, Brazil; ^7^ Post-Graduation Program in Health Sciences, Santo Amaro University (UNISA), São Paulo, Brazil; ^8^ Group of Cellular Oncology, Biodonostia Health Research Institute, San Sebastian, Spain; ^9^ CIBER On Frailty and Healthy Aging (CIBERfes), Institute of Health Carlos III, Madrid, Spain; ^10^ IKERBASQUE, Basque Foundation for Science, Bilbao, Spain; ^11^ Laboratory for the Evaluation of Sports Performance, Physical Exercise, and Health (LABMOV), Polytechnic of Guarda, Guarda, Portugal

**Keywords:** cardiovascular risk, frail older adults, blood markers, sarcopenia, platelets, Sustainable Development Goals 3

## Abstract

The effectiveness of Branched Chain Amino Acids (BCAAs) supplementation on enhancing exercise performance in both young and older adults remains a topic of debate. Recent research suggests that BCAAs combined with regular exercise might have an impact on human erythropoiesis, blood dynamics, and iron homeostasis. Given the increasing longevity of the global population, it is crucial to investigate the potential benefits of BCAA supplementation and regular exercise as non-pharmacological interventions for improving the overall health of frail older adults. To assess the influence of a 40-week multicomponent exercise intervention (MEP) combined BCCA supplementation on the haematological indicators of frail older adults (83–93 years old) residing in nursing homes. A prospective, naturalistic, controlled clinical trial employing an intervention-washout-intervention was conducted for this purpose. The study included four experimental groups: MEP plus BCAA supplementation (MEP + BCAA, *n* = 8), MEP only (*n* = 7), BCAA supplementation only (*n* = 7), and control group non exercising (CG, *n* = 13). Fried's physical frailty (PF) protocol was employed to stratify the participants. Additionally, the assessment included the evaluation of nutritional status, comorbidities, and anthropometric measurements. Among the several haematological markers examined, only mean cellular Haemoglobin Concentration (MCH) [F = 4.09; *p* < 0.03] and Mean Cell haemoglobin Concentration (MCHC) [F = 10, 323; *p* < 0,0001] showed significant effects of time group. Our findings demonstrate that a long-term intervention with BCAA plus MEP did not lead to significant alterations in the haematological profile. An 8-week withdrawal from interventions did not affect the frailty status in the MEP and MEP + BCAA groups, whereas the control group exhibited an increase in PF status. The findings, demonstrating the potential pro-immune effect and maintenance of MCH and MCHC levels, highlight the relevance of incorporating exercise and nutritional strategies to promote healthy aging. This study contributes to the achievement of the United Nations Sustainable Development Goals 3 (good health and well-being) and 10 (reduced Inequalities) for all.

## 1. Introduction

Frailty is characterized by a set of physical and cognitive impairments that are related to older adults living in nursing homes who tend to be have more difficult doing the essential daily tasks (1, 2). Europe has one of the highest rates of frailty among institutionalized older adults, with a prevalence of approximately 70%, indicating that frailty is a general characteristic of populations residing in long-term care (3). Despite its high prevalence and potential reversibility when detected and treated early, few studies have specifically focused on institutionalized older adults. This knowledge gap is significant considering the associated poor health outcomes and the urgency to address frailty in this population (4).

Although ageing is recognized as a natural and irreversible degenerative process, current data have shown that regular exercise, especially when accompanied by appropriate nutrition programs, is an efficient non-pharmacological intervention able to retard and (5), to some extent, prevent the fast progression of age-related physical and cognitive impairments. Exercise and nutritional supplements are widely established treatments and preventative measures for frail older adults (6). According to a recently published study (7), frail older adults should be referred to progressive Multicomponent Exercise Programs (MEP) that include resistance, balance, and aerobic training components, as well as multifactorial interventions involving supplementation (8). These interventions have been shown to effectively reduce and delay the onset of frailty while enhancing participants’ functional capacity and health-related quality of life.

According to literature, the multifactorial intervention programs is an innovative approach applied to older populations that includes progressive resistance strength training exercises, balance training for activities of daily living, cardiovascular exercises, and nutritional supplementation (7, 8). Although several dietary supplementation strategies have been investigated in recent years to mitigate the adverse effects of frailty and its related health outcomes (9, 10), the focus has primarily been on interventions incorporating Branched-Chain Amino Acids (BCAAs) and regular exercise (11, 12).

The supplementation with BCAAs, including leucine, valine, and isoleucine, has been suggested as one of the best low-cost strategies to prevent and mitigate motor and mental disabilities associated with aging and frailty (10, 13), particularly when combined with regular exercise programs (5, 7, 8). BCAAs are unique in this regard as they are primarily absorbed by skeletal muscle instead of the liver, making them special (14). The liver indirectly affects BCAA metabolism through the activity of the enzyme “branched-chain ketoacid dehydrogenase” (BCKD), which is known to be reduced in muscle tissue where BCAAs are oxidized (12). Additionally, the enzyme “branched-chain aminotransferase” is responsible for the reversible transamination of BCAAs (14, 15).

Studies have demonstrated that BCAAs play significant roles in enhancing protein synthesis by regulating the mammalian target of rapamycin **(**mTOR) pathway, serving as both an energy source and a precursor for other amino acids in muscle. BCAAs have also been shown to reduce muscle breakdown and increase protein synthesis (16). Regarding the mTOR mechanism, leucine has been shown to promote increased phosphorylation status of mTOR, inhibiting the action of the Tumour Suppressor Complex (TSC1/TSC2) complex (17). In addition to the administration of BCAAs, resistance exercise is characterized by an increase in growth factors such as insulin-like growth factor 1 (IGF-1), which activates phosphoinositide 3-kinase (PI3-Kinase) and, in turn, stimulates protein kinase B (PKB) expression (17). In turn, PKB can phosphorylate tuberous sclerosis complex 1 (TSC1), leading to a decrease in the production of the TSC1/TSC2 complex. Consequently, this cascade increase protein synthesis through the mTOR pathway and phosphorylation of the p70S6 kinase (18).

In the literature, more than 40 biomarkers of cardiovascular function have already been studied as potential predictors of frailty, although some controversy persists (19, 20). Several scales are currently used by gerontologists to monitor frailty, and the Fried frailty scale or Frailty Phenotype is one of the most accurate and widely used (21). Regarding blood biochemistry, specific blood components such as haemoglobin content (Hb), haematocrit percentage (%Hct), and platelet concentration have also been correlated with frailty and dementia (22). The relationship between lower levels of Hb and the decline of muscular strength and frailty has been analysed and validated by several clinical studies (22, 23). Furthermore, it is also known that higher %Hct or higher blood viscosity (directly associated with the rheostatic properties of blood) have been linked to a higher risk of cardiovascular diseases, insulin resistance, obesity, dyslipidaemia, hypertension, or even mortality (24–27).

Several studies have investigated the effects of exercise combined with BCAA protein supplementation in older adults, revealing significant benefits. The ingestion of leucine-enriched essential amino acids and carbohydrates following resistance exercise was found to enhance mTOR signalling and protein synthesis in human muscle (28). The another study focused on the effects of leucine and its metabolite β-hydroxy-β-methyl-butyrate on human skeletal muscle protein metabolism, showing positive outcomes (29). In a systematic review which examined the prevalence of sarcopenia in aging adults and interventions for its management, the importance of addressing muscle loss in older populations was emphasized (30).

Several studies investigating the effects of regular exercise training and BCAA supplementation in older adults have shown mixed results (31). For instance, a recent one with a same design found that BCAA supplementation did not improve muscle protein synthesis rates in older men during resistance exercise (32, 33). Similarly, a randomized controlled trial previous conducted reported no significant differences in muscle strength or physical function between the exercise plus BCAA supplementation group and the exercise-only group in older individuals (34). These contrasting findings emphasize the complexity of the topic. Besides a slight controversy, optimistic perspectives from recent and solid studies (3, 7) have fuelled the discussion about the efficacy of BCAA supplementation combined with regular exercise in improving the quality of life of older adults. In comparison to isolated supplements, a dietary strategy would therefore be more likely to yield a beneficial outcome in a higher percentage of older adults, especially when combined with MEP.

Therefore, the purpose of this study is to determine the impact of a 40-week multicomponent exercise program on haematological biomarkers and frailty scores in older adults living in residential care homes (RCH), with or without BCAA supplementation. Additionally, we hypothesize that participants' physical function and cardiovascular systems may be affected by the combination of MEP and BCAAs. Additionally, we hypothesize that the combination of MEP and BCAAs may impact participants' physical function and cardiovascular systems. Furthermore, we anticipate that there will be no significant changes in the overall status of the elderly during the 8-week period of the withdrawal of interventions.

## 2. Methods

### 2.1. Study design and settings

**Figure 1:**
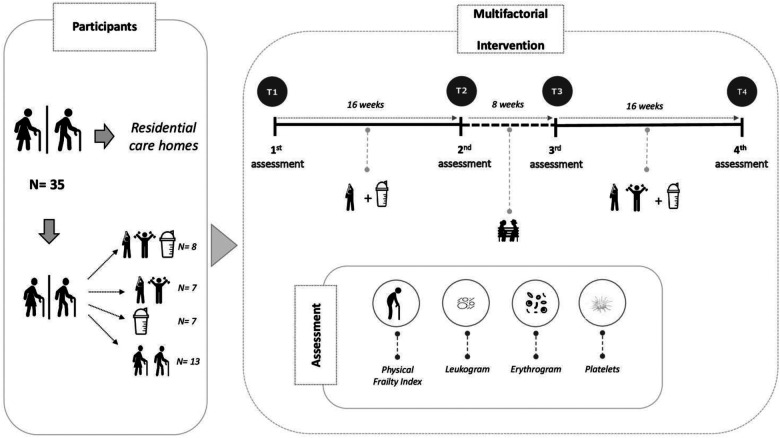
Timeline of the intervention study involving 4 intervention arms and the main indicators analyzed.

This is a four-phase prospective, naturalistic, controlled clinical trial (treatment vs. care) that includes a washout period (intervention-withdraw-intervention) with a four-arm experimental design (MEP + BCAAs, BCAAs, MEP, and CG). The study involved older adults living in RCH in the city of Coimbra, Portugal, who were 70 years old or older. The recruitment process for engaging institutions in this study has been previously outlined in studies published by our research group (8, 11). The first phase involved a baseline data collection (T1), followed by a 16-week intervention. In the second phase, data collection (T2) was conducted, and an 8-week washout period followed. In Phase 3, the intervention was resumed for another 16 weeks, with a third data collection. After the 16-week intervention period, the final data collection (T4) was conducted. The different stages of exercise and BCAA supplementation are presented in Figure 1.

### 2.2. Participants recruitment and allocation

Volunteers' eligibility, allocation, and experimental design have been fully described in previous studies conducted by our research group (8, 11). Briefly, out of the initially screened 50 volunteers, only 35 participants (83 ± 3 years) completed the 40-week intervention, as detailed in Figure 1. These volunteers were divided into the following groups: Multicomponent Exercise plus Branched Chain Amino Acid supplementation (MEP + BCAA, *n* = 8); Multicomponent Exercise (MEP, *n* = 7); Branched Chain Amino Acid supplementation only (BCAA, *n* = 7); and the non-exercise/no-supplementation control group (CG, *n* = 13). The guidelines of the Consolidated Standards of Reporting Trials (CONSORT) were followed for all procedures (35).

### 2.3. Data collection

This study encompasses four assessment points: T1 to T4. Once the experimental groups were established, baseline data was collected at T1, followed by a 16-week period of elastic-band strength training and/or BCAA supplementation, with a subsequent data collection at T2. This initial exercise intervention was succeeded by an 8-week washout period, concluded with a new data collection for detraining/washout analysis at T3. Finally, after the washout period, the strength exercise protocol was replaced by a MEP (including the former elastic band strength exercises, along with aerobic and balance exercises) for an additional 16-week period, culminating in a final data collection at T4 (see Figure 1).

### 2.4. Ethical statement

The exercise sessions and/or supplementation programs were voluntary for all participants, with a consent form signed by the administrators of the institution, the participants, or their legal representatives before the start of the intervention. This study, approved by the Ethical Committee of Faculty of Sport Sciences and Physical Education, University of Coimbra (reference number: CE/FCDEFUC/00282018), adhered to the Portuguese Resolution (Art. 4th; Law no. 12/2005, 1st series) on ethics in human research (36), and the Helsinki's Declaration (37). The clinicaltrials.gov registration number for this study is NCT04376463, complementing the newly published article by providing additional biochemical data.

### 2.5. Outcomes measures

Every evaluation was conducted between 10:00 and 11:45 AM. The initial session aimed to assess the biosocial and overall health status, cognitive profile, nutritional intake, physical condition, and physical frailty status of the volunteers. On the second day, non-fasting blood samples were collected and analysed.

#### 2.5.1. Biochemical analysis

Non-fasting blood samples were collected in the morning (between 10:00 a.m. and 11:00 a.m.) via venipuncture using EDTA-containing tubes at the four checkpoints (T1 to T4), following a 24–48 h period after the last training session. The collection took place in an isolated and quiet room after a 15-minute rest. Prior to blood collection, volunteers were instructed to abstain from alcohol and caffeine consumption and maintain their regular sleep patterns the night before. Immediate blood cell counting was conducted using an automated hematology analyzer, Coulter Act Diff (Beckman Coulter, USA). The following biochemical markers were analyzed: Red Blood Cell Count (Erythrogram), Mean Cell/Corpuscular Volume (MCV), Mean Cell Hemoglobin (MCH), MCHC Mean Cell Hemoglobin Concentration (MCHC), RDW Red Blood Cell Distribution Width (RDW), Haematocrit (HCT), Leukogram (White Blood Cell Count), Lymphocytes, Monocytes, Granulocytes, Platelets (PLT), and Mean Platelet Volume (MPV).

#### 2.5.2. Physical frailty Index

Fried's of Physical Frailty Phenotype were used (38). Volunteers were categorized as pre-frail if one or two criteria were met, frail if three or more were met, and robust if none of the five physical frailty signs were present (10): (i) Weight loss in volunteers was determined by self-reporting an unintentional weight reduction of at least 4 kilograms in the previous six months; (ii) Self-reported exhaustion was evaluated by agreement on questions number 7 (“I felt that everything I did was an effort”) and 20 (“I could not get going”) from the Center of Epidemiologic Studies for Depression scale (39); (iii) Hand-grip strength was assessed using a hand-held dynamometer (Lafayette 78,010, Sagamore, United States) and the better outcome from two trials was selected for scoring. Volunteers unable to perform the hand-held test and those in the lowest 20% were categorized as positive for that frailty index (40). The cut-off reference values for the hand-held test were ≥29 kg for males and ≥17 kg for female. (iv) Slowness was measured using the “15 feet (4.6 m) walking test”. Scores of times ≥7s for males and ≥6 s for females, based on the cut-off values of Fried's study population, were considered positive for slowness (38). Final scoring was based on the faster of the two trials; (v) Low physical activity levels were assessed using the short-version of International Physical Activity Questionnaire (41). The questionnaire classified physical activity into three categories: slightly active, very active, and inactive. Volunteers classified as inactive had a positive score for this physical frailty component. In this study, volunteers classified as frail (3 or more points) and pre-frail (2 points) were included in the intervention phase. In order to track the effects of the intervention over time, a continuous measure called the physical frailty total score (0 to 5 points) was used (38), and scores closer to 5 indicating more frailty condition.

#### 2.5.3. Nutritional and general health Status

The nutritional assessment and clinical and health status were conducted as previously described (11). Briefly, the diet was analyzed using specific tools such as photographic quantification of portions and food tables designed for the Portuguese population (42), following the methods of previous studies (43). Additionally, the Portuguese version of Mini Nutritional Assessment (MNA) questionnaire was administered (44). The clinical and health/illness status was evaluated using the Charlson comorbidity index (CCI), which took into account individual comorbidity registration, age, and gender to calculate the final CCI score (45). Anthropometric measurements included body mass (kg) and stature (cm). Body mass was measured using a portable scale (Seca®, model 770, Berlin, Germany) with a precision of 0.1 kg, while stature was determined using a portable stadiometer (Seca Body meter®, model 208, Berlin, Germany) with an accuracy of 0.1 cm. Body mass index (BMI) was calculated using the equation BMI = (body mass)/(stature)². Standardized procedures for these measurements were described in previous studies (46).

### 2.6. Full characterization of the multifactorial intervention

#### 2.6.1. Oral supplementation with BCAA

The BCAA powder mixture was composed of L-leucine (Leu), L-isoleucine (Ile), and L-valine (Val) in the mass proportion of 2:1:1 (MyProtein®, Cheshire, UK), accounting for 20 kcal per portion. The procedures related to supplement administration were implemented based on prior studies (47–49). An unflavored supplement was used to avoid ingestion preferences for specific flavors by the participants. The BCAA mixture was diluted in 200 ml of water and given immediately after the exercise sessions to the volunteers in the MEP + BCAAs group (47). The supplement dose was fixed at 0.21 g total BCAA/kg/session (48). The supplement dose was set at a total of 0.21 g between 09:00 and 11:30 a.m. in the BCAA group. The BCAA-supplemented (MEP + BCAAs and BCAAs) and BCAA-absent groups (MEP and CG) were split according to the proximity between the RCH, where the exercise programs were effectively applied. No documented interactions between the volunteers from the BCAA-supplemented and no-BCAA supplemented groups in our study.

#### 2.6.2. Multicomponent exercise program

Each session commenced with a brief five-minute warm-up consisting of range-of-motion exercises for the wrists, shoulders, hips, knees, and ankles, as well as walking. The exercise sessions (T1–T2 and T3–T4) were conducted twice a week, with a 36-hour break between sessions to allow for adequate rest and recovery. The exercise protocol adhered to the exercise prescription guidelines for older adults and the exercise periodization guidelines outlined by the American College of Sports Medicine (50). The exercise program for T1–T2 began with a two-week adaptation period, during which seven different exercises were performed using elastic bands (TheraBand®, Hygenic Corporation, Akron, OH, USA). Close supervision was provided to the volunteers during the initial two sessions to familiarize them with the equipment and make adjustments based on their perceived exertion level using the Rating Perceived Exertion scale, also called RPE-OMNI Scale (51). These introductory sessions aimed to educate the volunteers on proper training techniques, as well as assist them in selecting the appropriate elastic band color, length, and grip width.

After the initial two-week adaptation period, the exercise program's intensity was gradually increased. This involved adding two additional exercises, bringing the total to ten exercises, and incorporating different colored bands based on the OMNI table (35). To ensure safety, the volunteers' heart rate was monitored during the exercise sessions using Polar M200® (Polar Electro Oy, Kempele, Finland), randomly distributed among participants. The maximum HR (HR_max_) was calculated using a specific formula for the older population (52). A low to moderate intensity effort, around 50%–75% HRR, was attained as recommended by the American College of Sport Medicine (50).

$$\left[HR_{\max}\;=\;207\;(\mathrm{beats}\;\mathrm{per}\;\mathrm{minute},\;\mathrm{bpm})-0.7\;\times \mathrm{chronological}\;\mathrm{age}\right]$$

The exercise retraining program for T3–T4 also utilized the elastic bands, but it also included a walkability program, adding step exercises, coordination, and balance exercises as well as exercises that replicate physical activities used in daily life such as sitting and rising from the chair. Dumbbells and ankle/wrist weights were occasionally incorporated as well, forming a MEP that lasted for the same 16-week period (twice a week, alternating days, totaling 32 sessions).

#### 2.6.3. Washout and detraining period

During the (T2–T3) period of multifactorial intervention, the volunteers experienced an 8-week detraining and washout phase in which the supplementation and exercise regimens were halted. The objective of this phase was to investigate whether the physiological adaptations achieved during the initial intervention phase were maintained or if an 8-week cessation could lead to a reversal of the hematological, and physical fitness adaptations (53).

### 2.7. Statistical analysis

The Shapiro-Wilk test was used to verify the normality distribution of the collected data. Descriptive values are expressed as mean ± standard deviation (SD). To compare the mean changes over time between groups, repeated measures ANOVA (4 × 4 group vs. time) were performed. Bonferroni's post-hoc analysis was performed for paired comparisons of means when significant interactions were found in the dependent variables (Leukogram, Erythrogram, Platelets), and confidence intervals (CI: 95%) were reported when a significant effect was found. The level of significance was set at *p* < 0.05. All statistical analysis were done using IBM SPSS Statistics version 23.0 (Armonk, NY: IBM Corp, USA).

## 3. Results

Despite one isolated case of diarrhea (following the first BCAA supplementation), in which the participant quickly recovered and resumed the protocol without further issues, no other adverse events, injuries, or intervention-related complications were observed throughout the study. Authorized data obtained from RCH nurses revealed that 68% of the volunteers were using cardiovascular disease medications (atorvastatin/simvastatin). Please refer to Table 1 for more details.

**Table 1:** Characteristics of experimental and control groups at baseline.

	MEP + BCAA (*n* = 8)	MEP (*n* = 7)	BCAA (*n* = 7)	CG (*n* = 13)	*p* value
Age	80.00 ± 6.10	86.70 ± 4	84.20 ± 5.80	83.10 ± 5.40	0.14
Time in residential care (years)	3.60 ± 1.00	4.70 ± 1.40	4.50 ± 1.10	5.00 ± 1.00	0.06
Mini nutritional assessment (0–30 pts)	25.50 ± 2.20	24.00 ± 2.70	21.70 ± 2.80*	24.70 ± 1.80	0.02
Body mass index (kg/m^2^)	28.50 ± 5.10	28.70 ± 5.60	25.80 ± 3.10	30.20 ± 3.70	0.23
Stature (cm)	158.00 ± 0.10	150.00 ± 0.10	161.00 ± 0.10	155.00 ± 0.10	0.16
Comorbidity index (0–10 pts)	4.90 ± 1.10	5.30 ± 0.90	5.40 ± 1.10	4.90 ± 1.20	0.71
Physical Frailty index (0–5 pts)	2.00 (0.53)	2.71 (1.10)	3.00 (0.57)	2.16 (0.71)	0.40
Polypharmacy (per day)	5.60 ± 2.70	8.00 ± 4.00	6.10 ± 2.70	9.10 ± 3.50	
Hypertension	37.50% (3)	42.50% (3)	14.20% (1)	46.10% (6)	
Dyslipidemia/metabolic syndrome	50% (4)	28% (2)	40% (3)	38% (5)	
Heart failure	40% (3)	34% (2)	34% (2)	30% (4)	
Diagnosed mental disease	12% (1)	12% (1)	28% (2)	23% (3)	
Daily Individual Protein (gr/Kg/day)	1.42 ± 0.28	1.83 ± 0.44	1.48 ± 0.22	1.60 ± 0.23	
BCAA supplementation (per person/gr/day)	15.20 ± 3.00	–	14.20 ± 5.20	–	

* *p* < 0.05; MEP, multicomponent exercise program; M/SD, mean/standard deviation; BCAA, Branched Chain Amino Acids; CG, control group; BMI, body mass index; M ± SD, mean (standard and deviation); pts, points; Kg/m^2^, kilograms; cm, centimetres; One-way ANOVA was used to compare groups. except for Comorbidity index (fisher exact test).

### 3.1. Red blood cell count (erythrogram)

The values of the markers Erythrogram indexes for the different intervention groups before and after the multifactorial intervention are shown in Table 2. An important finding of the time*group interaction was observed for MCH (Mean Cell Hemoglobin) [F(df: 6.058, 34.32) = 4.200, *p* = 0.003]. Bonferroni comparisons revealed baseline differences between the ME + BCAA and BCAA groups (CI: −9.49, −0.04, *p* = 0.04). However, over time, significant changes were only observed in the ME + BCAA group between T1 and T2 (CI: −3.628, −0.806, *p* = 0.001), and in the CG (Control Group) between T2 and T4 values (CI: −3.12, −0.08, *p* = 0.03).

**Table 2:** Erythrogram indexes of the different intervention groups before-after multifactorial intervention (T1–T4).

Erythrogram	TIME	ME + BCAA	ME	BCAA	CG	Effect	F	Overall *p*
		M(SD)	M(SD)	M(SD)	M(SD)			
Erythrocytes 10^6^/mm^3^	T1	4.41 (0.37)	4.25 (0.28)	4.22 (0.41)	4.27 (0.77)	time	0.487	0.696
	T2	4.12 (0.58)	4.21 (0.34)	4.13 (0.33)	4.10 (0.67)			
	T3	4.29 (0.29)	4.13 (0.33)	4.30 (0.19)	4.10 (0.76)	time*group	0.435	0.910
	T4	4.32 (0.43)	4.10 (0.67)	4.17 (0.31)	4.22 (0.53)			
Hemoglobin (g/dl)	T1	11.80 (2.08)	11.90 (0.82)	13.00 (0.75)	11.67 (1.93)	time	0.360	0.783
	T2	11.80 (2.25)	12.00 (0.87)	12.23 (1.15)	11.35 (1.65)			
	T3	11.66 (1.86)	11.40 (0.86)	13.67 (0.57)	11.50 (2.07)	time*group	1.795	0.092
	T4	11.78 (1.48)	12.41 (1.23)	12.16 (0.90)	12.36 (1.55)			
MCV (μm^3^)	T1	87.80 (7.40)	91.18 (2.63)	93.70 (2.74)	91.90 (5.04)	time	0.024	0.995
	T2	87.50 (5.56)	91.30 (3.69)	93.20 (3.38)	92.10 (6.22)			
	T3	87.50 (7.52)	90.50 (3.29)	95.00 (4.96)	91.00 (6.52)	time*group	0.740	0.671
	T4	89.30 (1.16)	90.40 (3.73)	94.50 (5.33)	90.50 (6.27)			
MCH (pg)	T1	26.60 (3.34)	28.20 (1.21)	31.00 (2.09)	27.3 0 (1.39)	time	1.445	0.250
	T2	28.40 (2.14)	28.50 (1.18)	29.50 (0.76)	27.70 (1.62)			
	T3	27.10 (3.85)	27.70 (1.09)	31.00 (1.40)	28.10 (1.86)	time*group	4.200	0.003
	T4	27.10 (1.48)	29.80 (1.06)	29.13 (1.71)	29.38 (2.41)			
MCHC (g/dl)	T1	30.20 (1.52)	30.90 (0.66)	33.00 (1.28)	29.70 (0.56)	time	2.630	0.060
	T2	32.48 (0.48)	31.20 (0.64)	31.73 (0.47)	30.20 (0.65)			
	T3	30.90 (2.19)	30.60 (0.90)	32.66 (0.40)	30.90 (1.00)	time*group	10.323	0.000
	T4	30.40 (1.29)	33.00 (0.45)	30.80 (0.10)	32.40 (0.98)			
RDW (%)	T1	13.10 (0.87)	13.60 (0.93)	13.30 (0.37)	13.90 (0.88)	time	0.413	0.744
	T2	13.70 (1.49)	13.20 (0.47)	12.40 (0.25)	13.60 (0.98)			
	T3	15.00 (4.96)	13.10 (0.32)	12.80 (0.30)	13.60 (0.95)	time*group	1.057	0.410
	T4	13.30 (0.79)	13.40 (0.45)	13.00 (0.30)	13.80 (0.90)			

T, time of data collection; M/SD, mean/standard deviation; MEP, multicomponent exercise program; BCAA, branched chain amino acids; CG, control group; MCV, mean cell/corpuscular volume; MCH, mean cell hemoglobin; MCHC, mean cell hemoglobin concentration; RDW, red blood cell distribution width; HCT, Hematocrit.

Significant effects of time*group interaction were found for MCHC (Mean Cell Hemoglobin Concentration) [F(df: 9, 51) = 10.323, *p* < 0.001]. Bonferroni *post hoc* analysis showed that, in a similar way for MCH results, the baseline values of MCHC parameter in BCAA group was higher than the ME + BCAA group (CI: −5.218, −0.749, *p* = 0.006). At T2, CG showed lower MCHC values in comparison to all other groups (*p* < 0.001, *p* = 0.035, *p* = 0.011, for ME + BCAA, ME, and BCAA groups, respectively). While for T3 no differences emerged between groups, at the last follow-up (tT4) ME group presented the higher MCHC levels in comparison to ME + BCAA group (CI: 1.017, 4.183, *p* = 0.001) and BCAA group (CI: 0.261, 4.139, *p* = 0.021). MCHC levels also differ between the groups CG and ME + BCAA in the same time point (CI: 0.417, 3.583, *p* = 0.009). In addition, over time, significant differences were found between T1 and T2 (CI: −3.685, −1.182, *p* < 0.001), T2 and T3 (CI: 0.69, 3.064, *p* = 0.037), as well as T2 and T4 (CI: 0.839, 3.328, *p* = 0.001) in the ME + BCAA group. Furthermore, in the ME group, the MCHC values at T4 were significantly higher compared to T1 (CI: 0.689, 3.477), T2 (CI: 0.489, 2.978), and T3 (CI: 0.975, 3.658; *p* < 0.05 for all). The BCAA group also showed a significant difference in MCHC between T1 and T4 (*p* < 0.05), while no significant changes were observed at other checkpoints in the same group. Additionally, the CG exhibited an increase in MCHC over time, with the highest values obtained at T4 compared to T1 (CI: 1.239, 4.027), T2 (CI: 0.955, 3.445), or T3 (CI: 0.158, 2.842) (*p* < 0.05 for all).

### 3.2. White blood cell count (leukogram)

The values of the markers Leukogram indexes for the different intervention groups before and after the multifactorial intervention are shown in Table 3. No significant changes for time and time*group interactions were found for other parameters of RBC (T1-T4).

**Table 3:** Leukogram indexes of the different intervention groups before-after multifactorial intervention (T1–T4).

		ME + BCAA	ME	BCAA	CG	Effect	F	Overall *p*
	TIME	M(SD)	M(SD)	M(SD)	M(SD)			
Lymphocytes (×10^3^/ul)	T1	2.04 (0.58)	1.93 (0.42)	1.26 (0.20)	1.93 (0.45)	time	0.131	0.940
	T2	1.90 (0.44)	2.06 (1.44)	1.33 (0.58)	1.82 (0.35)			
	T3	2.02 (0.57)	1.81 (0.52)	1.23 (0.30)	1.96 (0.97)	time*group	0.557	0.825
	T4	1.73 (0.46)	1.81 (0.64)	1.60 (0.78)	1.75 (0.31)			
Monocytes (×10^3^/ul)	T1	0.45 (0.19)	0.31 (0.14)	0.23 (0.11)	0.48 (0.14)	time	0.778	0.526
	T2	0.48 (0.24)	0.36 (0.16)	0.33 (0.25)	0.38 (0.29)			
	T3	0.45 (0.27)	0.43 (0.21)	0.26 (0.20)	0.68 (0.40)	time*group	1.787	0.096
	T4	0.70 (0.41)	0.21 (0.98)	0.70 (0.34)	0.30 (0.17)			
Granulocytes (×10^3^/ul)	T1	5.50 (2.07)	5.30 (1.55)	4.30 (1.73)	5.31 (1.70)	time	0.517	0.677
	T2	5.00 (1.38)	5.98 (3.62)	3.23 (0.90)	4.90 (1.99)			
	T3	5.41 (0.87)	4.58 (0.87)	3.50 (1.31)	4.75 (1.47)	time*group	0.964	0.481
	T4	5.66 (2.33)	4.96 (1.02)	3.66 (1.43)	5.05 (1.88)			

T, time of data collection; MEP, multicomponent exercise program; BCAA, branched chain amino acids; CG, control group; M(SD), mean/standard deviation.

### 3.3. Platelets

The values of the markers Platelets indexes for the different intervention groups before and after the multifactorial intervention are shown in Table 4. Regarding platelets, no significant effect of time or time*group interactions were observed in any of the evaluated indexes (*p* > 0.05).

**Table 4:** Platelets indexes of the different intervention groups before-after multifactorial intervention (T1–T4).

		ME + BCAA	ME	BCAA	CG	Effect	F	Overall *p*
	TIME	M(SD)	M(SD)	M(SD)	M(SD)			
PLT (×10^3^u/L)	T1	209.00 (88.54)	184.83 (52.53)	166.0 (30.80)	213.66 (39.70)	time	0.712	0.549
	T2	232.66 (126.27)	206.50 (25.10)	145.33 (17.24)	216.16 (47.40)			
	T3	233.66 (91.18)	193.83 (50.66)	166.33 (36.11)	212.00 (53.00)	time*group	1.017	0.440
	T4	251.16 (79.02)	187.50 (49.80)	117.00 (80.43)	215.50 (43.44)			
MPV (fl)	T1	7.91 (0.48)	8.75 (0.96)	8.60 (0.36)	9.38 (0.62)	time	0.922	0.437
	T2	7.90 (0.87)	8.43 (0.82)	8.53 (0.2)	8.96 (0.73)			
	T3	8.10 (1.04)	8.60 (0.90)	8.73 (0.66)	8.76 (0.72)	time*group	1.043	0.420
	T4	7.93 (1.05)	8.51 (0.80)	8.93 (0.49)	8.76 (0.43)			

PLT, platelets; MVP, mean platelet volume; T, time of data collection; MEP, multicomponent exercise program; BCAA, branched chain amino acids; CG, control group; M(SD), mean/standard deviation.

### 3.4. Physical frailty

**Figure 2:**
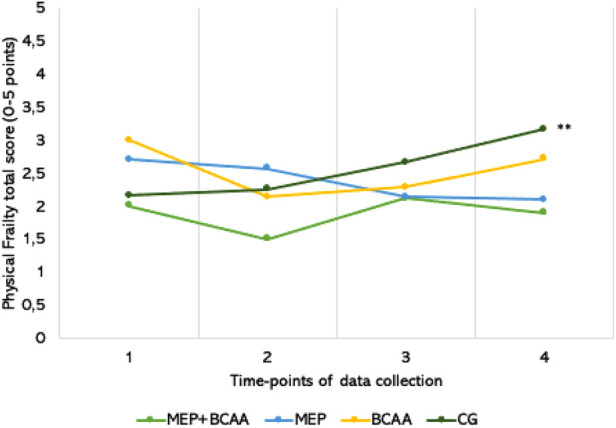
**p < 0.001; the total frailty scores-variation of all intervention groups in the 40-week of multifactorial intervention, that consisting of 16 weeks of intervention (T1–T2), an 8-week withdrawal period (T2–T3), and another 16 weeks of intervention (T3–T4).

A significant interaction between time vs. group was observed for the frailty index (F = 3.799, *p* = 0.001). No significant differences between groups were found at T1, T2, and T3. However, at the final follow-up (T4), the CG exhibited higher levels of frailty compared to both the MEP + BCAA and BCAA groups (*p* = 0.008 and *p* = 0.012, respectively). As depicted in Figure 2, over time, the BCAA group exhibited a notable reduction in the frailty index from T1 to T2 (*p* = 0.005). In contrast, the Control Group experienced a slight increase in frailty during each follow-up, leading to a significant difference between T1 and T4 (*p* < 0.001).

## 4. Discussion

Taken into account that the goal of this study was to determine the impact of a 40-week MEP on hematological biomarkers and physical frailty scores in older adults living in residential care homes (RCH), supplemented or not with BCAA, the results obtained corroborated our initial hypothesis that the MEP, particularly when combined with BCAA supplementation, could lead to improvements in hematological biomarkers, as well as in physical frailty.

In fact, our findings demonstrated that intervention with MEP was able to ameliorate the overall frailty levels of the participants, indicating that this exercise training program, especially when combined with BCAAs supplementation, effectively can contribute to reducing physical frailty in older adults. Additionally, the results concerning MCH and MCHC parameters allow us to putatively suggest that the interventions applied in the present study have positively impacted the erythrocyte concentration and hemoglobin levels, in a similar way as formerly reported when the exercise training was combined with BCAA supplementation (54). These results support the notion that a comprehensive approach incorporating both exercise and nutritional interventions can yield significant benefits in mitigating physical frailty and improving hematological parameters in older adults.

### 4.1. Hematological changes

In relation to the hematological parameters assessed here, it was possible to observe significant differences in the time*group analysis only in the MCH and MCHC values. In this respect, it is worth pointing out that the baseline differences found in these parameters between the BCAA group and ME + BCAA group could be attributed to the nutritional status since a similar result was observed in the Mini Nutritional Assessment (MNA). In accordance with the literature, some factors, such as aging, chronic inflammation, and nutritional status can influence the red blood cell distribution (55, 56), which includes the MCH and MCHC parameters. At this point, it is paramount to mention that the statistically significant differences in the baseline nutritional status between these groups could be associated with the randomization process of the volunteer groups. It is well-known that these variations in nutritional status can not only reflect diverse dietary habits and lifestyle factors according to socioeconomic status (57), but also influence the volunteers' overall health and physical condition, which contributes to the development of frailty (58). Therefore, addressing and exploring these differences could be useful to provide valuable insights into the intricate relationship between nutrition, exercise, and frailty in older adults.

Beyond the baseline significant differences, specifically, the MCHC values found in the 3 groups submitted to a type of intervention were higher than the values found in the control group at the T2 time point. In a general way, the increased MCHC values can represent an elevation of the concentration of hemoglobin inside the red blood cells (RBC), or, alternatively, that hemoglobin is found outside of the RBC due to its destruction. Since the normal range of MCHC is around 29 to 35 g/dl, our results demonstrated that all volunteer groups maintained their MCHC level in normal conditions during the study period. Therefore, we can suggest that the increase of MCHC at the T2 time point in the MEP, MEP + BCAA, and BCAA groups can indicate that both the exercise training program and BCAA supplementation were able to improve the bioavailability of oxygen to tissues. In fact, it is worth citing that regular endurance training can accelerate the removal of the oldest RBCs, which induces the production of new RBCs (59). Thus, in line with these pieces of information, the MEP applied in the present study, by including aerobic training, could putatively contribute to the elevation of MCHC values by favoring the RBCs removal and, consequently, the generation of new cells with more hemoglobin quantity in order to improve oxygen-carrying capacity leading to the better tissue oxygenation. In a different way, older adults submitted to a MEP intervention during acute hospitalization did not present alteration in the RDW levels (56).

Despite the effect of BCAA supplementation on hematological parameters is still scarce in the literature, it was reported that BCAA supplementation increased not only the RBCs number and hematocrit, as well as the hemoglobin levels in outpatients with liver cirrhosis (60). Particularly in the older adult population, it was documented that an improvement in the oxygen-carrying capacity by RBCs was attributed to the combined effect of exercise and BCAA supplementation (15, 16). These findings align with the notion that the MEP, when combined with BCAA supplementation, can exert a beneficial influence on hematological parameters, contributing to the overall well-being of older adults (30, 32).

In an interesting way, a formerly research demonstrated that BCAA supplementation improved muscle mass in cirrhotic patients with sarcopenia (61). Additionally, another study showed that BCAA supplementation improved the liver frailty index in frail compensated cirrhotic patients (62, 63). These findings support the notion that BCAAs have a positive impact on muscle mass and functional outcomes in older adults. Additionally, our group also showed that exercise interventions combined with BCAA supplementation can modulate inflammatory markers and enhance muscle strength and power (11, 48).

Based on the report that there is a positive association between hemoglobin concentration and physical performance, the importance of optimizing hematological parameters for maintaining overall physical function in older adults is emphasized (64). Future studies should consider exploring the long-term effects of MEP and BCAA supplementation on various health outcomes, including innovative myokines as biomarkers related to immune function. These findings have practical implications for the development of exercise and nutritional interventions aimed at improving physical frailty and hematological biomarkers in older adults, ultimately enhancing their overall well-being and quality of life.

Concerning the results related to the different time points assessed here (intragroup analysis), since the alterations observed in both MCH and MCHC parameters were, in a general way, similar between the volunteer groups, we can putatively suggest that these findings were attributed to the seasonal variation. In fact, it has been demonstrated that the hematological parameters are closely influenced by the seasonal variation in different populations, including athletes and healthy individuals (65–67). Interestingly, it was evidenced that hematocrit and hemoglobin levels decrease in the summer due to the hemodilution (65, 66), and/or increase in the winter due to the hemoconcentration (68).

Platelets are the lowest cellular components in blood. The activation of platelets for further degranulation and aggregation is fundamental for blood haemostasias and are mediated by several specific platelet-stimulating mediators, such as *α*-Granules (69–71). Nevertheless, no significant changes in platelet distribution were observed here with exercise and/or BCAA supplementation of older individuals. However, studies already published by other groups showed significant differences between the active and the inactive groups regarding platelet scores. Sample size, the experimental groups (anthropometric values, age, inactivity, etc.), and logistic specificities of our study could explain such discrepancies.

The statistically significant differences in the nutritional profile at baseline between the study groups have some implications for the results interpretation. These variations can impact the absorption and utilization of BCAA supplementation, potentially affecting its effectiveness in combination with MEP (72). Moreover, differences in nutritional status may influence the participants' overall health and physical condition, contributing to frailty and confounding the intervention effects (58). It is crucial to acknowledge these baseline differences when discussing the study findings and their implications. Additionally, the variations in nutritional profile reflect diverse dietary habits and lifestyle factors according the socioeconomic status (57), which can significantly influence health outcomes and interact with the interventions under investigation.

### 4.2. Impact on frailty status

Frailty in the MEP group showed a tendency to decrease over time, indicating the positive effects of exercise. Significant reduction in frailty was observed only during the initial 16 weeks of treatment with BCAA supplementation. Similarly, the MEP + BCAA group exhibited a similar pattern, further confirming the beneficial impact of BCAA supplementation in combination with exercise. These findings underscore the effectiveness of the exercise program, particularly when combined with BCAA supplementation, in reducing physical frailty in older adults residing in RCH (73). Institutionalization is a psychosocial event experienced by individuals during different stages of their lives, providing social and health protection (74). The British Geriatric Society emphasizes the importance of regular procedures and agreed-upon measurements in social and health care relationships with older adults to screen for frailty, including sarcopenia. These recommendations underscore the significance of adopting an active lifestyle, and dietary protein intake, as a strategy to mitigate the negative consequences of physical frailty and prevent its progression to more severe conditions (75, 76).

The outcomes of our study align with previous research highlighting the potential of non-pharmacological interventions, such as exercise programs, to address physical frailty and sarcopenia in older individuals (77). In the same direction, the study involving the same population on chair-based exercise programs in institutionalized older women highlighted the positive effects of such interventions on salivary steroid hormones, disabilities, and frailty changes, further supporting the benefits of exercise programs in improving health outcomes in older adults (78). Based on these findings, we suggest that exercise, BCAA supplementation, or their combination could potentially ameliorate frailty in institutionalized older individuals. It is noteworthy that the control group was the only group that experienced a notable decline in frailty status, highlighting the importance of interventions in preventing frailty progression. Institutionalization provides an opportunity for health protection. Non-pharmacological interventions, like exercise and diet programs, are effective in addressing frailty. Prevention is crucial to halt frailty progression.

### 4.3. Limitations, novel and directions for future studies

The study had limitations including a small sample size, lack of diversity, and a relatively short intervention duration. The small sample size may limit the generalizability of the findings and statistical power. The focus on frail older adults in nursing homes may not represent the broader population, suggesting the need for more diverse samples. The 40-week intervention duration might not have been long enough to observe significant changes in hematological markers, indicating the importance of longer intervention periods for better understanding the effects of BCAA supplementation and exercise. The novelty of the study lies in the combination of BCAA supplementation and MEP. This unique approach contributes to the existing literature by investigating the effects of this combination on hematological indicators in frail older adults. The study adds to the current understanding of the potential benefits of BCAA supplementation and exercise by examining their combined effects, providing valuable insights into the efficacy of this novel intervention strategy.

Our findings align with real-world data reflecting the challenges faced in nursing homes, where participants exhibit various impairments, comorbidities, and motivational obstacles. Conducting a 40-week controlled study with this population is subject to additional limitations, such as unforeseen factors like mortality or changes in nursing home residency. Future studies should focus on long-term follow-up assessments to determine the sustained effects of BCAA supplementation and exercise interventions on hematological markers and physical frailty in older adults. Additionally, mechanistic investigations are needed to explore the underlying physiological mechanisms involved. Comparisons with other interventions and investigations into individual differences in treatment response should also be considered to enhance our understanding and personalize interventions for older adults.

### 4.4. Practical applications

The practical purposes of our study are twofold. Firstly, the combination of BCAA supplementation and MEP can be considered as a potential non-pharmacological intervention for improving the hematological indicators and physical frailty of older adults residing in nursing homes. This suggests that healthcare professionals and caregivers can incorporate these interventions into the care plans of frail older adults to enhance their overall well-being. Secondly, our study highlights the importance of long-term follow-up assessments and personalized interventions that consider individual differences to optimize the efficacy of BCAA supplementation and exercise programs in this population. This knowledge can guide future research and clinical practice in promoting healthy aging and improving the quality of life for older adults in residential care settings.

## 5. Conclusion

Our study provides valuable insights into the effects of BCAA supplementation and a MEP on hematological indicators and physical frailty in older adults residing in nursing homes. Although the long-term intervention did not result in significant changes in the hematological profile, the combination of BCAA supplementation and MEP demonstrated potential pro-immune effects and maintained certain hematological markers. These findings contribute to the existing literature by examining the effectiveness of these interventions in frail older adults, emphasizing the importance of incorporating exercise and nutritional strategies for promoting healthy aging. Moreover, our study aligns with the Sustainable Development Goal 3, as it addresses the objective of ensuring good health and well-being for older adults. Personalized interventions, long-term follow-up assessments, and consideration of individual differences can optimize the efficacy of interventions, improving health outcomes and well-being for frail older adults in residential care settings.

## Data Availability

The original contributions presented in the study are included in the article/Supplementary Material, further inquiries can be directed to the corresponding authors.
