# Supplementary material for: Empowering frail older adults: multicomponent elastic-band exercises and BCAA supplementation unleash physical health and preserve haematological biomarkers
Source: Front Sports Act Living. 2023 Aug 30;5:1171220. doi: 10.3389/fspor.2023.1171220 (PMC10502309; doi:10.3389/fspor.2023.1171220)
Supplement: Supplementary file 1 [file Table1.docx]

Supplementary Material

Empowering Frail Older Adults: Multicomponent Elastic-band Exercises and BCAA Supplementation Unleash physical health and Preserve Haematological Biomarkers

Adriana Caldo-Silva*, Guilherme E. Furtado*, Matheus Uba Chupel, Rubens Vinícius Letieri, Rafael Santos Neves, Fábio Direto, Marcelo P. Barros, André L. L. Bachi, Ander Matheu, Faber Martins, Alain Massart, Ana Maria Teixeira,

*** Correspondence:** Corresponding Author: Adriana Caldo-Silva dricaldo@gmail.com Supplementary Data

Supplementary Material should be uploaded separately on submission. Please include any supplementary data, figures and/or tables.

Supplementary material is not typeset so please ensure that all information is clearly presented, the appropriate caption is included in the file and not in the manuscript, and that the style conforms to the rest of the article.

# Supplementary Figures and Tables

**Table 1.** Example of elastic-band exercises session applied in phase 1

| **Warm-up** 5 minutes RPE 1-3 | | | | | | **Progression** | **Weeks** | **Intensity (colour)** |
| --- | --- | --- | --- | --- | --- | --- | --- | --- |
| **Exercises (8-10)** | **Sets** | **Repetitions** | **Cadence** | **Interval** | **RPE** | 2x10 | 2 | Yellow |
| Front squat | 2-3 | 10-20 | 2:3 | 30-45 seconds | 4 to 6 | 3x20 | 2 | Yellow |
| Chair unilateral hip flexion | 2-3 | 10-20 | 2:3 | 30-45 seconds | 4 to 6 | 3x10 | 2 | Red |
| Chair Bench over row (with flexion) | 2-3 | 10-20 | 2:3 | 30-45 seconds | 4 to 6 | 3x20 | 2 | Red |
| Chest Press (stand and/or chair) | 2-3 | 10-20 | 2:3 | 30-45 seconds | 4 to 6 | 3x10 | 2 | Green |
| Standing (or chair) reverse fly | 2-3 | 10-20 | 2:3 | 30-45 seconds | 4 to 6 | 3x20 | 2 | Green |
| Shoulder Press/twist arm position | 2-3 | 10-20 | 2:3 | 30-45 seconds | 4 to 6 | 3x15 | 2 | Blue |
| Chair (or stand) frontal total raiser | 2-3 | 10-20 | 2:3 | 30-45 seconds | 4 to 6 | 3-4x10-15 | 2 | Blue |
| Biceps arm curl (stand and/or chair) | 2-3 | 10-20 | 2:3 | 30-45 seconds | 4 to 6 |  |  |  |
| Chair Overhead triceps extension | 2-3 | 10-20 | 2:3 | 30-45 seconds | 4 to 6 |  |  |  |
| **Cooling down** 5 minutes RPE 1-2 | | | | | |  |  |  |

Notes: Rate of Perceived Exertion

**Table 2**- Multicomponent Exercise Program (ME)

| **Exercises (8-10)** | **Sets** | **Repetitions** | **Cadence** | **Interval** | **RPE** |
| --- | --- | --- | --- | --- | --- |
| Front squat | 2-3 | 10-20 | 2:3 | 30-45 seconds | 4 to 6 |
| Chair unilateral hip flexion | 2-3 | 10-20 | 2:3 | 30-45 seconds | 4 to 6 |
| Chair Bench over row (with flexion) | 2-3 | 10-20 | 2:3 | 30-45 seconds | 4 to 6 |
| Chest Press (stand and/or chair) | 2-3 | 10-20 | 2:3 | 30-45 seconds | 4 to 6 |
| Standing (or chair) reverse fly | 2-3 | 10-20 | 2:3 | 30-45 seconds | 4 to 6 |
| Shoulder Press/twist arm front position | 2-3 | 10-20 | 2:3 | 30-45 seconds | 4 to 6 |
| Chair (or stand) frontal total raiser | 2-3 | 10-20 | 2:3 | 30-45 seconds | 4 to 6 |
| Biceps arm curl (stand and/or chair) | 2-3 | 10-20 | 2:3 | 30-45 seconds | 4 to 6 |
| Chair Overhead triceps extension | 2-3 | 10-20 | 2:3 | 30-45 seconds | 4 to 6 |
| **Circuit training** |  |  |  |  |  |
| Walking around the room | 2-3 | 3 minutes |  | 30-45 seconds | 4 to 6 |
| Balance/ agility exercise | 2-3 | 3 minutes |  | 30-45 seconds | 4 to 6 |
|  |  |  |  |  |  |

Notes: Rate of Perceived Exertion
